# Dexamethasone Associated ST Elevation Myocardial Infarction Four Days after an Unremarkable Coronary Angiogram—Another Reason for Cautious Use of Steroids: A Case Report and Review of the Literature

**DOI:** 10.1155/2016/4970858

**Published:** 2016-07-18

**Authors:** Mohamed Shokr, Ahmed Rashed, Kusum Lata, Ashok Kondur

**Affiliations:** ^1^Internal Medicine Department, Detroit Medical Center/Wayne State University, Detroit, MI 48201, USA; ^2^Cardiology Department, Detroit Medical Center/Wayne State University, Detroit, MI 48201, USA

## Abstract

Drug induced myocardial infarction is a known entity with different forms of steroids linked to coronary artery disease (CAD) either through promoting its traditional risk factors, inducing coronary spasm, or by other unidentified mechanisms. Dexamethasone is known to promote an atherogenic and hypercoagulable state. We report a case of a 75-year-old woman who had ST elevation myocardial infarction (STEMI) associated with dexamethasone use just 4 days following an angiogram showing minor luminal irregularities.

## 1. Introduction

ST elevation myocardial infarction is a substantial health problem with estimated 30-day mortality between 2.5 and 10% [1]. Nontraditional risk factors play a significant role in more than 50% of CAD cases [2]. More than 150 drugs were reported as possible causes of acute MI. Betamethasone, methylprednisolone, and dexamethasone were among 39 drugs highlighted as prime suspects that can cause acute MI [3]. We report a case of STEMI associated with dexamethasone use 4 days following a coronary angiogram with only minor luminal irregularities.

## 2. Case Presentation

**Figure 1:**
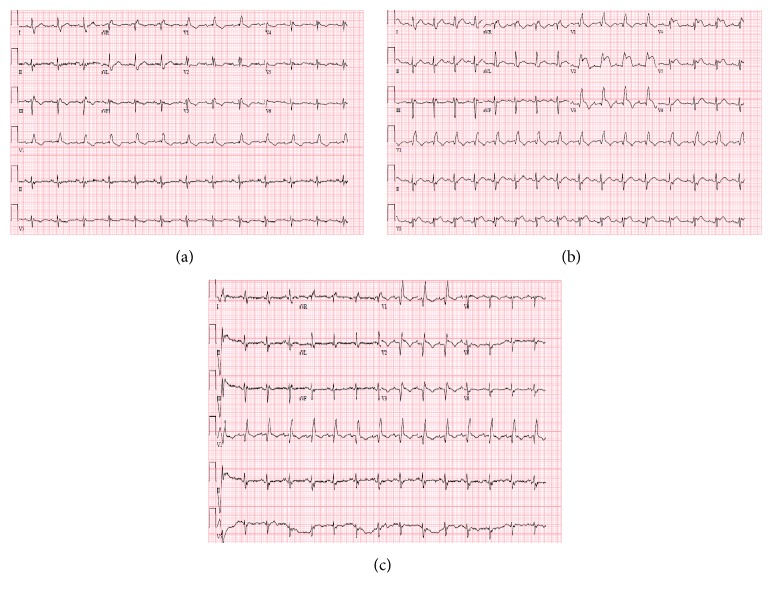
(a) Baseline EKG with RBBB. (b) EKG showing ST elevation in leads V2–V5. (c) EKG showing resolution of the ST elevation following LAD intervention.

Our patient is a 75-year-old African-American woman with a history of diabetes mellitus, hypertension, hyperlipidemia, and four prior cerebrovascular accidents with mild right sided residual weakness and ventral hernia status after surgical repair. She has no prior history of smoking, alcohol use, illicit drug use, or family history of premature coronary artery disease. She initially presented with shortness of breath, rhinorrhea, and cough productive of yellowish sputum and tested positive for influenza A for which she was started on oseltamivir. On her second day of hospitalization she reported mild retrosternal chest pain accompanied with a Troponin I elevation up to 0.3 ng/mL with an unchanged EKG showing right bundle branch block (RBBB) (Figure 1(a)). She received Aspirin, Clopidogrel, and Heparin drip in addition to Diltiazem and Atorvastatin as treatment for a NSTEMI.

**Figure 2:**
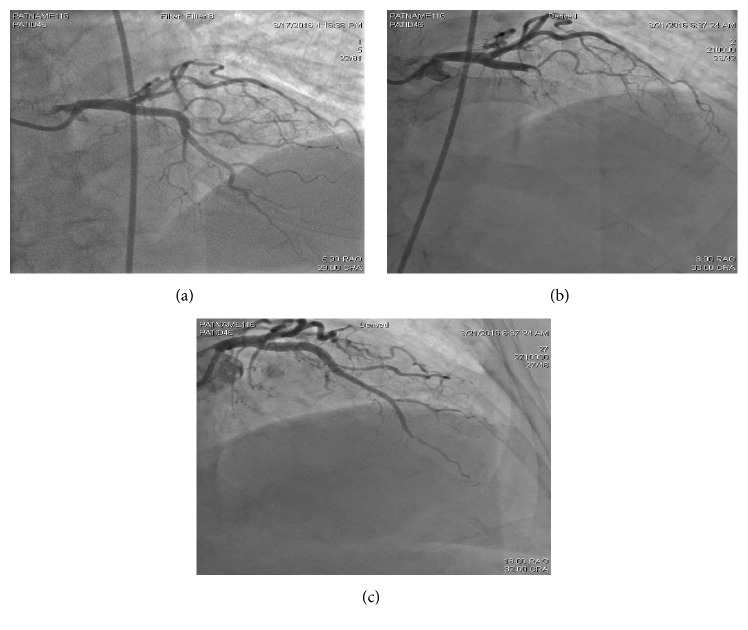
(a) First left heart catheterization showing LAD with minor luminal irregularities. (b) Second left heart catheterization (4 days later) showing the mid-LAD lesion. (c) LAD after intervention.

On the following day, she underwent a coronary angiogram revealing only minor luminal irregularities and no significant CAD (Figure 2(a)). Two days later, she underwent nasopharyngolaryngoscopy for progressive dysphonia that showed inflammatory changes of true and false vocal folds, mild granulation changes of the subglottis, and pachydermia. This was deemed secondary to her upper respiratory tract infection and the resulting acute tracheobronchitis. Subsequently, she was started on intramuscular dexamethasone 10 mg Q8 hours.

One day later, she complained of severe retrosternal chest pain with a blood pressure of 90/65 mmHg, heart rate of 95 bpm, and oxygen saturation of 95% on room air. Cardiovascular exam was unremarkable revealing regular heart rate and normal S1 and S2 with no appreciated murmurs, rubs, or gallop. She had no JVD and clear lungs on auscultation. Distal pulses were well felt without appreciated lower extremity edema. The EKG however showed ST elevation in the anterior leads, V2 to V5 (Figure 1(b)). Bedside echocardiogram revealed a left ventricular ejection fraction (EF) of 30–35% and regional wall motion in the form of apical dyskinesis and severe hypokinesis in the mid to apical anteroseptal, anterior, apical inferior, inferoseptal, and lateral segments. Of note, the pre-MI echocardiogram showed a normal EF with no appreciated regional wall motion abnormalities. The Troponin I level was 0.1 ng/mL (cutoff value of 0.2 ng/mL). She was immediately transferred to the catheterization lab where the angiogram surprisingly revealed a filling defect, likely a thrombus, occluding the midsegment of the LAD (Figure 2(b)). Subsequently, a 2.5 × 18 mm drug eluting stent was deployed with pre- and postballoon dilation resulting in TIMI III flow (Figure 2(c)). Eptifibatide was started in addition to Ticagrelor, Aspirin, Deltiazem, and Atorvastatin. The chest pain resolved shortly after the intervention and the EKG returned to baseline (Figure 1(c)).

Blood work revealed a platelet count of 461 k/cumm, hemoglobin of 10.5 mg/dL, and white blood cell count of 9 k/cumm. Homocysteine level was mildly elevated (15.5 micromoles/L, normal range 3.2–10.7). High sensitivity CRP was elevated at 40.9 mg/L. Factor VIII activity (325%, normal range: 63–150), factor XI activity (211%, normal range: 71–124), and thrombin antithrombin complex (13.7 ng/mL, normal range: 0.7–3.2) were all increased while factor VII activity was normal. Fibrinogen level and protein C and S activity were normal. Cardiolipin antibody, JAK2 V617F mutation, and Factor V Leiden were all negative.

## 3. Discussion

Besides impacting traditional risk factors for CAD such as hypertension (HTN), glucose intolerance, obesity, and hyperlipidemia (HLD), glucocorticoids influence vascular functions, atherogenesis, and remodeling following ischemia or intravascular injury. This influence is mediated by both glucocorticoids and mineralocorticoid receptors and is modified by 11b-hydroxysteroid dehydrogenase enzyme local metabolism of glucocorticoids [4].

Dexamethasone was shown to promote an atherogenic and hypercoagulable state through different mechanisms. In high doses, it increases Von Willebrand Factor (VWF) which is a prothrombotic marker and increases the platelet activator P-selectin. Moreover, short term use of dexamethasone is associated with increased levels of Fibrinogen, VII, VIII, and XI [5, 6].

In 2014 Okumura et al. reported 3 cases of steroid induced coronary spasm [7]. This might be explained by the fact that, in a dose dependent manner, cortisol treatment decreases ATP-induced intracellular calcium mobilization, which in turn decreases nitrate, nitrite, and nitric oxide release [8]. Similarly, glucocorticoids downregulate cyclooxygenase-1 gene expression and subsequently suppress the production of the vasodilator prostacyclin; meanwhile they increase the synthesis of thromboxane which is a vasoconstrictor [9]. There is also evidence that elevated serum cortisol levels might sensitize the coronary vasoconstricting responses through Rho-kinase activation [10]. Additionally, it reduces endothelial nitric oxide synthase mRNA stability and inhibits the synthesis of tetrahydrobiopterin, which is an important cofactor of endothelial nitric oxide synthase [11].

Interestingly, Okumura hypothesized that corticosteroid-induced vasospastic angina might be underestimated due to being mistakenly confused with its known side effect of epigastric discomfort. Wei et al. reported that, among patients using high dose glucocorticoids (>7.5 mg/day), the relative risk for cardiovascular events occurrence was 2.56 [12].

In 2007, Varas-Lorenzo et al. performed a cohort study with nested case-control analysis to estimate the risk of acute myocardial infarction (AMI) associated with the use of oral corticosteroids by dose and duration. The study included 4795 hospitalized cases of AMI or CAD deaths and randomly sampled 20,000 controls and frequency matched by sex, age, and calendar year.

The adjusted OR for AMI in current users of oral corticosteroids compared to nonusers was 1.42 (95% CI: 1.17–1.72). The risk during the first 30 days of use (OR = 2.24; 95% CI: 1.56–3.20) was greater than with longer duration (OR = 1.22; 95% CI 0.98–1.52). The risk was more pronounced (OR = 2.15; 95% CI 1.45–3.14) among users of prednisolone equivalent doses >10 mg/day. The study concluded that there is a small increased risk of AMI with oral corticosteroid use with a greater risk observed among users of high corticosteroid dose [13].

In 2013, Coloma et al. identified 163 drugs reported to cause acute MI in various case reports, out of which 39 had a more probable association with MI including betamethasone, prednisolone, and dexamethasone (RR (95% confidence interval) 3.2 (2.9, 3.5), OR 1.9 (1.7, 2.2), and IRR 5.4 (4.1, 7.2)). Of note, 285 excess cases representing patients on dexamethasone who had an AMI however with other more plausible causes were identified [3]. All the previous mechanisms might explain a possible “spasm-thrombosis” sequence that occurred in our patient in the setting of her long standing other risk factors including diabetes mellitus, hypertension, and hyperlipidemia as mentioned. Interestingly, two prior cases reported angiographically normal coronaries in androgen using athletes who ended up having a coronary occlusion [14]. We are including a comparison between six prior cases of AMI associated with steroid use shedding the light on their different clinical scenarios and other preexisting risk factors for coronary artery disease (Table 1).

## 4. Conclusion

Different forms of steroids have been linked to CAD either through promoting its traditional risk factors or by other unidentified mechanisms. In most reported cases of steroid-associated AMI, the temporal relation was the main factor suggesting causality. We believe that, with the wide use of corticosteroids for a variety of indications, physicians should be aware of this possible association. Cautious use should be considered particularly in patients with other risk factors for CAD until there is a larger and more convincing pool of evidence.

## Figures and Tables

**Table 1:** Comparison between six other cases of MI associated with steroid use.

Case	Ferenchick and Adelman [14]	Yildirim et al. [15]	Arslan et al. [16]	Takamatsu et al. [17]	Owecki and Sowiński [18]	Poorzand et al. [2]
Age (years)	37	64	20	79	66	23

Risk factors	Family history of CAD	HLD	Smoking (7 years)	Bortezomib use	Smoking HLD	

Gender	Male	Male	Male	Female	Female	Male

Steroid type	Nandrolone-decanoate, Boldenone, testosterone-cypionate, Stanozolol, and veterinary oxandrolone	Prednisolone	Methylprednisolone	Dexamethasone	Methylprednisolone	Dexamethasone

Indication	Anabolic steroids (weightlifting)	Idiopathic intracranial HTN (papilledema)	Anaphylaxis	Multiple myeloma	Graves	Anabolic steroids (wrestling)

Route	Intramuscular & oral	Oral	Intravenous	Intravenous	Intravenous	Intramuscular

Dose	200 mg/week for 16 weeks 1.5 cc q3 days/16 weeks 1.5 cc q4 days/16 weeks 1.5 cc q3 days/16 weeks 50 mg daily for 16 weeks	40 mg daily	40 mg	NA	1 gm daily	NA

Duration of use	Intermittent for 7 years and then 16 weeks before the event	One month	7 minutes	5 days	5 days	6 months

Possible confounders			Hypotension secondary to anaphylaxis	Bortezomib use		

Acute coronary syndrome type	STEMI	NSTEMI and then STEMI few days later	STEMI	STEMI	NSTEMI	STEMI

EKG	ST elevation, II, III, and aVF	ST elevation, II, III, and aVF; ST depression, I, aVL	ST elevation, I, aVL	ST elevation, aVR; ST depression, I, II, and aVF V2–6	NA	ST elevation, I, aVL

Echo	Normal	Normal	EF 35% apical & posterolateral wall motion abnormality	EF 68% posterior & anterolateral akinesia	Anteroseptal akinesia	EF 35% apical, midanterior, anteroseptal akinesia

Left heart catheterization findings	Normal coronaries (3 days following tissue plasminogen activator)	RCA: slow flow and then PDA total occlusion & LAD slow flow	Normal coronaries 10 days later (normal IVUS) (treated with Aspirin & Heparin)	LM/LCX significant lesions LAD mod stenosis	LAD total occlusion RCA critical stenosis	Nonobstructive CAD (4 days after streptokinase)

Complications						LV thrombus
